# Tracking and Treating Fungal Contamination in Indoor‐Growing Barley Sprouts

**DOI:** 10.1111/1758-2229.70161

**Published:** 2025-07-31

**Authors:** Dedong Kong, Mengdi Dai, Ziran Ye, Yu Luo, Xuting Chen, Xiangfeng Tan

**Affiliations:** ^1^ Institute of Digital Agriculture Zhejiang Academy of Agricultural Sciences Hangzhou China

**Keywords:** barley sprout, fungal contamination, indoor farming, mycobiome, ozone water, seed endophytes

## Abstract

The industrialised production of barley sprouts is a nutritional replenish for livestock, whereas it is being threatened by fungal contamination derived from the closed and humid environment. This study investigates the fungal communities in barley seeds and sprouts and explores the utilisation of ozone water as a mould control method. In barley seeds of 10 cultivars, *Alternaria alternata* , *Phoma epicoccina* and *Fusarium cerealis* were the most abundant fungal species and varied between barley cultivars. A significant transformation in fungal communities after seed germination was observed, featured by the shifted community structure and a significant decline of alpha diversity in the eight‐day sprouts. *Arthroderma vanbreuseghemii*, *Fusarium cerealis* and *Candida quercitrusa* were identified to proliferate in eight‐day barley sprouts. Ozone water treatment was effective in suppressing fungal contamination including *A. vanbreuseghemii* and several *Fusarium* spp. Among the volatile organic compounds, the abundance of 3‐Octanone isomers was significantly reduced by ozone water treatment, suggesting its potential role as a volatile marker for monitoring mould outbreak. Our research emphasises cultivar‐specific fungal profiles in the production of barley sprouts and proposes ozone water as an effective control measure to ensure the safety of barley sprouts.

## Graphical abstract

This study identifies shifts in fungal communities during barley sprouting, with Arthroderma vanbreuseghemii and Fusarium cerealis dominating in eight‐day sprouts. Ozone water effectively suppresses pathogenic fungi, whereas 3‐octanone volatile compounds indicate mould contamination, offering a potential biomarker for rapid detection.

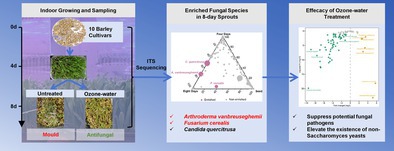

## 1 Introduction

Barley ( *Hordeum vulgare* L.) is a globally recognised staple cereal crop and serves dual purposes as a source of human nutrition and a primary constituent in animal feed, specifically in the form of germinated seeds known as barley sprout. These sprouted barley seedlings present a robust nutritional profile, encompassing high levels of proteins, fibres, vitamins and minerals, thereby constituting an indispensable component of livestock diet (Fazaeli et al. 2012; Gebremedhin et al. 2015). It has been shown that animals fed barley sprouts exhibit improved growth rates, nutrient utilisation and feed conversion ratios compared to conventional diets (Devendar et al. 2020). Since the production process of barley sprout is resource‐efficient, it allows year‐round, high‐yield production with minimal water and land usage (Soufan 2023). Moreover, the controlled environment of hydroponic systems contributes to environmental sustainability by utilising alternative water sources and minimising strain on grazing lands (Devendar et al. 2020). Nevertheless, the quality and safety of these sprouts are perpetually jeopardised by fungal contamination (Chethan et al. 2021), which can precipitate severe economic losses and pose grave health risks to animals.

Fungal contamination in barley sprout is originated from diverse sources. Environmental factors are instrumental in the propagation of fungi, with conditions such as elevated humidity, warm temperatures and the presence of decomposing organic matter fostering optimal environments for fungal growth (Carmona et al. 2008). Diverse fungal contaminations have been reported to outbreak in indoor agricultural systems, including *Rhizopus oryzae* in barley (Dai et al. 2024), *Bremia lactucae* in lettuce (Garibaldi et al. 2012) and the airborne *Cladosporium* species in multiple‐use greenhouses (Li and Lamondia 2010; Wu and Wong 2022). A study integrating cases of fungal disease outbreaks from global public databases has concluded that humidity is a critical factor influencing the occurrence of fungal diseases, including fungal genus *Puccinia*, *Fusarium* and the fungal‐like (i.e., oomycete) genus *Phytophthora*, which are among the most commonly reported causative agents of plant diseases (Romero et al. 2022). In addition to these harmful pathogens, seeds also harbour endophytic fungi, whose roles can range from beneficial to potentially harmful depending on environmental conditions and host interactions (Turkington et al. 2002; Carmona et al. 2008). For instance, under certain conditions, some endophytic fungi may shift from being symbiotic to becoming parasitic, leading to significant impacts on crop health. In the context of sprouting systems, it is crucial to understand how these endophytes behave. Although some endophytes enhance plant resistance to pathogens and improve nutrient uptake, others may produce toxins (Cavaglieri et al. 2009) or become pathogenic when environmental stressors such as high humidity or temperature fluctuations occur. Therefore, understanding the dynamics between endophytic fungi and their hosts during the sprouting process is essential for developing effective management strategies to mitigate potential risks.

The genetic constitution of barley cultivars can exert a significant impact on their susceptibility to fungal contamination. In the field, seed traits such as seed hulls (Gangwar et al. 2019), biochemical profiles (Martin et al. 2018) and accumulation of phenolic compounds (Kazimierczak et al. 2020) have been correlated with fungal disease resistance. Additionally, cultivar‐specific endophytic communities can modulate colonisation by pathogenic fungi (Sapkota et al. 2023; Pacheco‐Moreno et al. 2024). However, most existing studies focus on the barley‐fungus interactions in the field, where soil microbiota, fluctuating microclimates and agronomic practices influence outcomes. In contrast, indoor barley sprouting systems rely primarily on seed‐borne defences, such as hull morphology, intrinsic antimicrobial metabolites and resident endophytes, since soilless, high‐humidity conditions may favour different fungal assemblages than those encountered in the field. Therefore, to select a suitable barley cultivar to produce barley sprouts, it is necessary to elucidate the interactions between barley genotypes and fungal contamination.

In response to fungal contamination, an array of preventive and control measures have been implemented. These measures span from the strategic employment of fungicides to the application of biological control agents, such as beneficial microbes capable of outcompeting or inhibiting the growth of pathogenic fungi (Walters et al. 2012; Xi et al. 2022). However, microbial‐based solutions often require precise strain selection, maintenance of microbial viability and may be less predictable under high‐humidity indoor conditions (Walters et al. 2012). In contrast, ozone water has attracted attention due to its proven broad‐spectrum antifungal efficacy, rapid degradation into oxygen and negligible residual toxicity, making it ideal for food‐grade environments (Gonçalves 2016; Romeo‐Oliván et al. 2022). Ozone water has demonstrated broad‐spectrum activity against a wide range of fungal species in the prevention of grapevine trunk diseases (Romeo‐Oliván et al. 2022), seed sterilisation (Kang et al. 2015) and food storage (Gonçalves 2016). Additionally, ozone water can be conveniently integrated into sprouting workflows without requiring major structural or procedural changes. This operational simplicity, coupled with its environmental compatibility and effectiveness against spores and hyphae, makes ozone water a compelling choice for fungal management in barley sprout production systems.

Fast tracking methods for mould outbreak are also eagerly needed since visual discrimination is often lagging. High‐throughput microbial sequencing is a reliable method and has been incorporated in biocontamination detection in crop production and the food industry. Moreover, advances in volatile organic compounds (VOCs) also provide a promising early‐detection method for fungal contamination, since mould outbreak is usually parallel with noticeable odour (Liu et al. 2018; Mota et al. 2021). However, VOC sensors are largely diagnostic and not yet widely applied for active control in real‐time. Therefore, they should be integrated with more reliable intervention strategies, such as ozone water treatment. Considering barley sprouts are used for animal feed and finally affect human health through the food chain, efficient and reliable fungal control measures should be continuously developed and improved.

This study endeavours to probe further into the issue of fungal contamination during the production of barley sprouts. By employing high‐throughput sequencing techniques, we aim to furnish a comprehensive characterisation of the fungal communities present in the seeds and sprouts of 10 barley cultivars. The results pinpointed the major fungal contaminants during barley sprouting in different barley cultivars and verified the effectiveness of fungal treatment with ozone water, suggesting the necessity of selecting suitable cultivar selection and control methods for better quality of barley sprouts for animal feed.

## 2 Methods

### 2.1 Plant Growth Conditions

**FIGURE 1:**
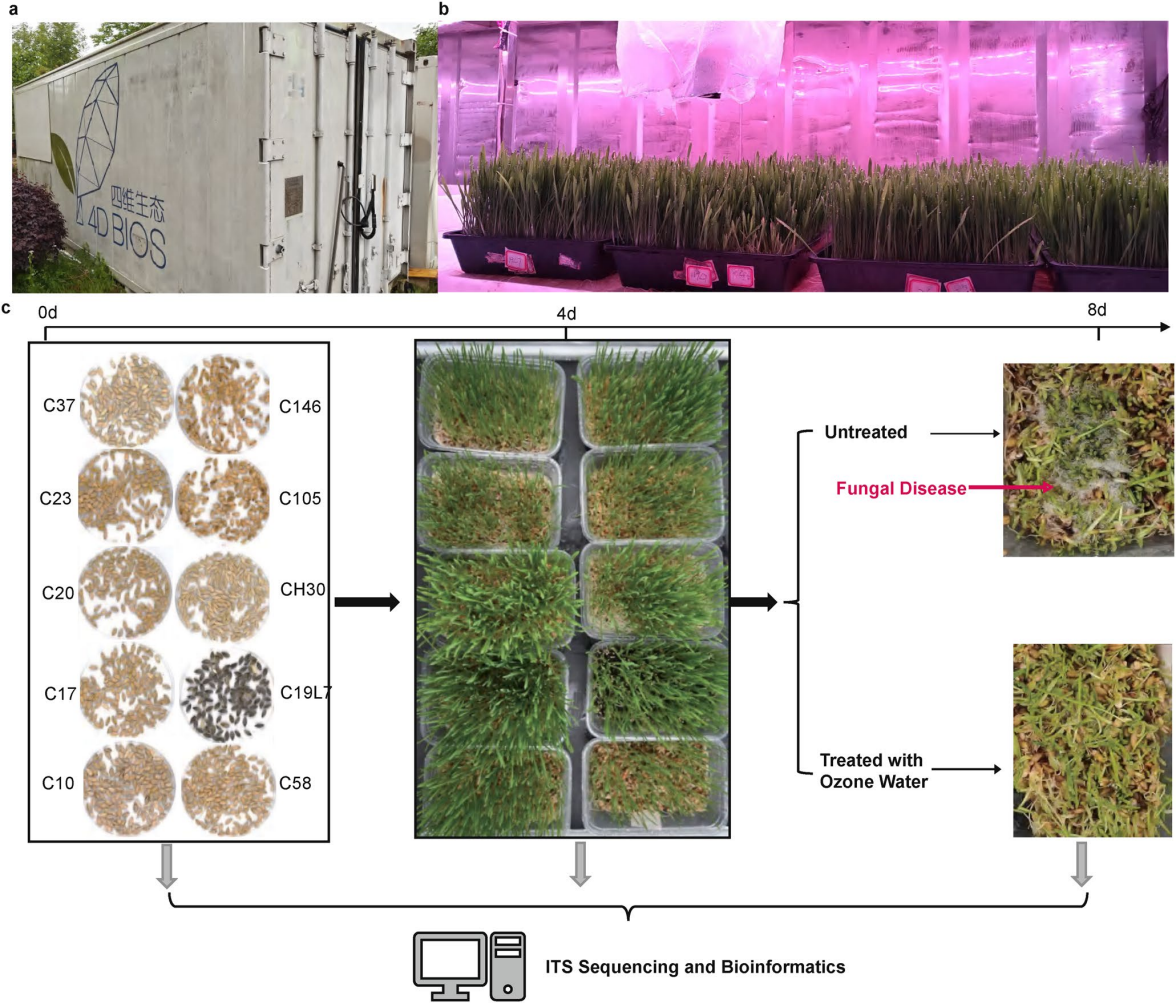
Experimental setup. Outside (a) and inside, (b) view of the containerised growth chamber for barley sprout. (c) Workflow of the experiments. Seeds of 10 barley cultivars were germinated in rectangular plastic containers. After 4 days of growth, half of the containers were treated with ozone water and the others were left untreated. Barley seeds, four‐day sprouts, and eight‐day sprouts (ozone‐water‐treated and untreated) were sampled separately for ITS sequencing and subsequent bioinformatic analyses.

A 20 m^2^ growth chamber was used to produce barley sprouts with an automated controlled environment (4D‐Bios Ltd., Hangzhou, China) (Figure 1a,b). Prior to the start of the experiment, the growth chamber was disinfected daily for 1 h using a portable OPPLE UV‐C germicidal lamp (Foshan, China) for three consecutive days. The recirculating nutrient solution system was sanitised with 100 ppm sodium hypochlorite (NaClO) solution by circulating the disinfectant through the system for 30–60 min. Following disinfection, the entire system was thoroughly rinsed with distilled water to remove residual chlorine. The temperature was 22°C and the relative humidity was 85%. These specific conditions were determined through preliminary optimisation trials, which enhanced germination rate and biomass yield while shortening the overall production cycle. The total experimental duration was 8 days. Barley seeds were germinated without light during the first 3 days, and a light intensity of ~40 μmol m^−2^ s^−1^ and a photoperiod of 18/6 h light/dark was set up in the remaining 5 days.

Ten different cultivars of barley bred by Zhejiang Academy of Agricultural Sciences were selected for the study, including two hulless (absence of hull adhering to grain) cultivars of ‘Mi 105’ and ‘Mi 146’ (C146), and eight hulled (presence of hull adhering to grain) cultivars of ‘Zhepi 10’ (C10), ‘Zhepi 17’ (C17), ‘Zhepi 19 L7’ (C19L7), ‘Zhepi 20’ (C20), ‘Zhepi 23’ (C23), ‘Zhepi 37’ (C37), ‘Zhepi 58’ (C58) and ‘Hua 30’ (C30) (Figure 1c). Seeds were surface‐sterilised in 95% ethanol for 30 s, followed by treatment with 2% (w/v) NaClO for 3–5 min. They were subsequently rinsed five times with sterilised deionised water. Seeds were germinated and grown in square plastic units with a top size of 17.3 cm × 11.5 cm, a bottom size of 13.5 cm × 8.3 cm and a depth of 5.3 cm. In each of the unit, 50 g of barley seeds were tucked in the bottom. Ten replicates were grown for each of the cultivars, resulting in a total of 100 individual growth units for the study. Throughout the experiments, water was sprayed every 4 h, automatically regulated by an electronic controller positioned above the growth units, whereas excess water was drained through pre‐drilled holes at the bottom of the plastic containers.

On the fourth day after germination, five growth units for all cultivars were treated with ozone water (2 ppm, pH 8.4) produced by an ozone water generator. Ozone water (50 mL) was manually sprayed on barley sprouts twice a day for 5 days, whereas untreated growth units received 50 mL of sterilised deionised water as a control. The concentration of ozone water was determined based on a previous study demonstrating the effectiveness of 2 ppm ozone water in suppressing fungal contamination during barley sprouting (Dai et al. 2024).

### 2.2 Experimental Sampling

Before germination, three replicates of barley seeds (5 g) were sampled for each of the 10 cultivars. Seeds were surface sterilised in advance with 0.3% NaOCl for 1 min and frozen by liquid nitrogen. After seed germination for 4 and 8 days, respectively, three replicates of barley sprouts were sampled for each of the 10 cultivars. Additionally, three replicates of sprouts for each of the 10 cultivars treated with ozone water were also sampled on the eighth day after germination. For the sprouts, shoots were removed by a sterilised scissor and the roots and ungerminated seeds were mixed and sampled for 5 g. Preliminary trials indicated that fungal colonisation was predominantly concentrated at the root–seed interface, whereas shoot tissues showed negligible fungal growth. Shoots were therefore excluded to focus on the compartment with highest fungal load and to ensure sample consistency.

### 2.3 PCR Amplification

Samples were stored at −80°C before DNA extraction. Genomic DNA is extracted from the samples, and the quality and quantity of the extracted DNA are assessed using 1% agarose gel electrophoresis. The primers of ITS1F (5′‐CTTGGTCATTTAGAGGAAGTAA‐3′) and ITS2R (5′‐GCTGCGTTCTTCATCGATGC‐3′) with barcodes are synthesised for the targeted sequencing region. To ensure accuracy and reproducibility in downstream analysis, low‐cycle PCR amplification is employed, maintaining consistency in the number of cycles across all samples. A pre‐experiment is conducted on representative samples to determine the minimum cycle number needed for adequate product concentration. The PCR reaction utilises TransStart Fastpfu DNA Polymerase (TransGen Biotech, Beijing, China) and ABI GeneAmp 9700 thermocycler (Life Technologies, Carlsbad, CA, USA). Each sample undergoes triplicate reactions, with PCR products combined and further analysed by 2% agarose gel electrophoresis. PCR products are then recovered using the AxyPrepDNA Gel Extraction Kit (Axygen Biosciences, Union City, CA, US). PCR products are quantified using the QuantiFluor‐ST Blue Fluorescence Quantitation System (Promega, Madison, WA, USA), ensuring the amount meets the sequencing requirements for each sample.

### 2.4 Library Preparation and Sequencing

The process includes attaching ‘Y’‐shaped adapters, purifying against adapter dimers using magnetic beads, enriching the library templates through PCR, and denaturing with sodium hydroxide to create single‐stranded DNA fragments. Sequencing is performed on the Illumina MiSeq platform (Illumina, San Diego, CA, USA) and raw sequence data is demultiplexed based on barcode sequences. Fastq files are generated for each sample, containing paired‐end reads. Quality control measures include trimming reads with low‐quality ends (quality score < 20 within a 50 bp window), merging paired‐end reads with a minimum overlap of 10 bp, allowing up to 0.2 mismatches in the overlap region, and filtering out reads with ‘N’ bases or primer mismatches. Sequences are oriented and assigned to their respective samples according to barcode and primer sequences. In summary, 9,884,784 raw reads and 2,975,319,984 total bases (bp) were obtained.

### 2.5 Bioinformatics Processing

**FIGURE 2:**
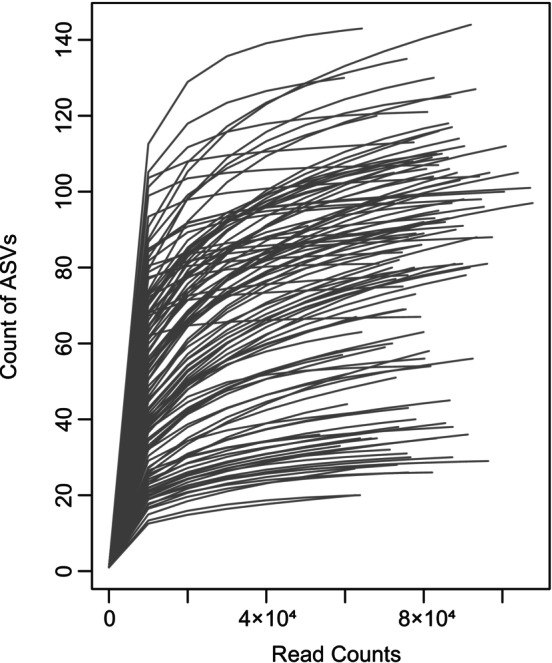
Rarefaction curve of samples at the level of ASVs. The lines in the figure represent the plotted number of observed fungal ASVs against the number of sequenced reads.

Raw DNA sequences were processed with Usearch11 (Edgar 2013). Paired‐end sequences underwent quality filtering using the ‘*fastq_filter*’ command and were then merged using the ‘*fastq_mergepairs*’ command. Unique reads were picked out with the ‘*‐fastx_uniques*’ and default options. Next, we clustered unique sequences into amplicon sequence variants (ASVs) with the ‘*‐unoise3*’ command (Edgar 2016). The ASV table was generated with the ‘‐otutab’ command and the read counts were normalised with the ‘*‐otutab_rare*’ command with the ‘*‐sample_size*’ option set as 50,557 (the minimum filtered read counts for the samples). For taxonomic lineage assignment, we utilised the ‘‐sintax’ command referencing the RDP Warcup training set v2 (18k sequences) database with a confidence threshold of 0.8. The phylogenic tree of ASVs was built using FastTree software. A rarefaction curve was built plotting the number of observed fungal ASVs against the number of reads, showing a sufficient coverage of ASVs for the ITS sequencing (Figure 2). The abundance table of fungal ASVs were aggregated at different taxonomic levels for further analyses, including species and genus levels.

### 2.6 Detection of VOCs

Four replicates were selected for eight‐day C105 barley sprouts treated with ozone water or untreated, respectively. The cultivar C105 was chosen because it displayed the heaviest mould symptoms during preliminary trials. Preliminary observations indicated that fungal colonisation and the corresponding impact of ozone treatment were concentrated in roots, with minimal visible infection on shoots. Therefore, we focused VOC profiling on roots to capture the most relevant signals. To determine the volatile compounds in samples using GC‐IMS technology (FlavourSpec, G.A.S. mbH, Dortmund, Germany), 5 g of barley roots were placed in a 20 mL headspace vial. The sample was then incubated at 45°C for 15 min before injecting 500 μL into the system. The GC‐IMS conditions include a 20‐min analysis time, using an MXT‐5 chromatography column (15 m × 0.53 mm) with a column temperature of 60°C and nitrogen as the carrier/drift gas at an IMS temperature of 45°C. The automatic headspace sampling conditions are set with an injection volume of 500 μL, an incubation time of 15 min, an incubation temperature of 40°C, an injection needle temperature of 85°C and an incubation speed of 500 r/min. The analysis of volatile compounds in different samples was performed using the VOCal software with built‐in databases for qualitative analysis. For comparative analysis, the Gallery Plot plugin within VOCal software was employed. This tool facilitates the generation of VOC ‘fingerprints’ by visualising the signal intensity of detected compounds across multiple samples in a two‐dimensional matrix. Each row in the Gallery Plot represents a sample, whereas each column corresponds to a specific VOC, enabling rapid visual comparison of compound abundance patterns. This approach allows for semi‐quantitative assessment of VOCs and highlights differences in volatile profiles among samples.

### 2.7 Statistical Analysis

Statistical analyses were performed in RStudio Build 353 and R 4.2.1. The phyloseq v1.42.0 package was used for analysing the ASV table. The read counts of each sample were normalised in the DESeq2 v1.38.3 package with the ‘DESeq’ function. PERMANOVA was employed to examine the effects of barley cultivar and sampling timepoints on the composition of fungal composition with the ‘adonis’ function in the vegan v2.6–4. Principal coordinate analysis (PCoA) was performed to compare the beta diversity between samples and treatments based on the weighted UniFrac distance matrices with the ‘ordinate’ function in the phyloseq package. Shannon indices were calculated for the fungal alpha diversity with the ‘plot_richness’ in the phyloseq package. One‐way ANOVA was performed followed by tukey's test in the agricolae v1.3–7 R package; bonferroni corrections were performed for the comparisons of VOC signal intensities by dividing *α* = 0.05 with the number counts of detected compounds, resulting a significance level of *p* < 0.0007. Correlations between fungal species were performed in the psych v2.3.12 R package with Spearman method and Bonferroni correction for *p* values. Proliferation of fungal species (mean relative abundance > 0.01% in all samples) was analysed in edgeR v3.40.2 and ggtern v3.4.2 R packages with a negative binomial generalised linear model (glmFit) and a likelihood ratio test; significant results were defined as *p* < 0.05 after Bonferroni corrections (Tan et al. 2022). Heatmaps for fungal relative abundance were visualised with the pheatmap v1.0.12 package. To demonstrate the effectiveness of ozone‐water treatment, log_2_‐transformed fold change values were used to describe the relative changes in normalised read counts of fungal species between ozone‐water‐treated and untreated barley sprouts.

## 3 Results

### 3.1 Seed Fungal Communities in 10 Barley Cultivars

**FIGURE 3:**
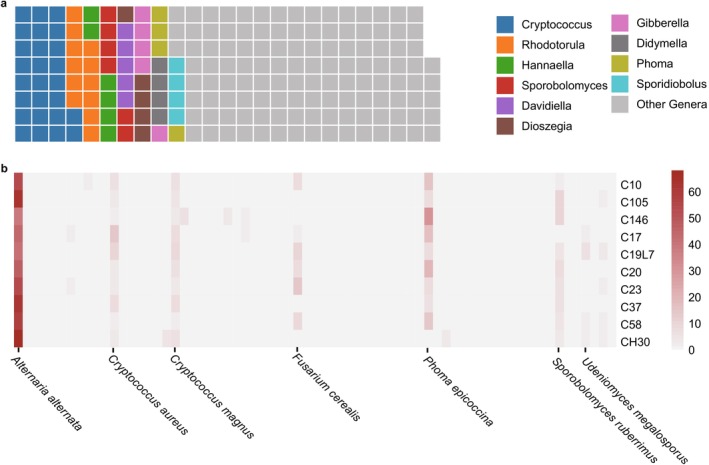
Diversity and composition of seed fungal communities. (a) Waffle plot of the observed fungal genera. Each block represented a fungal species and the different colours represented fungal genera. For clarity purposes, only the genera with the greatest numbers of fungal species were presented and the other genera were labelled as grey colour. (b) Relative abundance (%) of fungal species in 10 barley cultivars. For clarity purposes, only the species with the highest relative abundance were labelled.

In the seeds of the 10 examined barley cultivars, ITS amplicon sequencing resulted in 358 ASVs, belonging to 99 genera and 197 species. Among these genera, *Cryptococcus* (26 species) and *Rhodotorula* (12 species) showed the greatest number of species (Figure 3a), which are common non‐Saccharomyces yeast species. The fungal composition at the species level significantly differentiated between the 10 barley cultivars (PERMANOVA, *p* = 5 × 10^−4^, *R* ^2^ = 0.57), which was characterised by the varied abundance of *Alternaria alternata* , *Phoma epicoccina*, *Cryptococcus magnus* and *Fusarium cerealis* compared between barley cultivars, and these species also showed high relative abundance in barley seeds (Figure 3b).

### 3.2 Comparisons of Fungal Communities Between Barley Seeds and Sprouts

**FIGURE 4:**
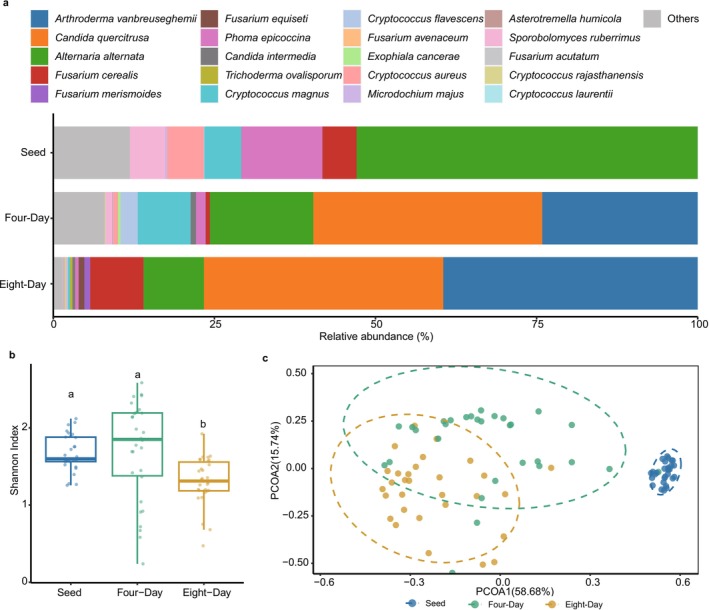
Comparisons of fungal communities between barley seeds and sprouts. (a) Fungal composition in the seed, four‐day sprouts and eight‐day sprouts of 10 barley cultivars. The 20 most abundant fungal species were shown and other species were aggregated into the ‘Others’ group. (b) Comparison of alpha diversity (Shannon index) between seed, four‐day sprouts and eight‐day sprouts. Letters indicated posthoc comparisons between treatments (Tukey's test). (c) Principal coordinate analysis (PCoA) of microbiomes in seed (*n* = 30), four‐day sprouts (*n* = 30) and eight‐day sprouts (*n* = 30) in 10 barley cultivars.

Fungal composition distinctly differentiated between barley seeds and the sprouts. *Arthroderma vanbreuseghemii* and *Candida quercitrusa* showed extremely low relative abundance of less than 0.01% in barley seeds but showed spikes in barley sprouts on both fourdays and eightdays after germination (Figure 4a). In contrast, the relative abundance of many fungal species drastically reduced after seed germination, including *Alternaria alternata* and *Phoma epicoccina*. Moreover, several *Fusarium* spp., which are common fungal pathogens, increased their abundance after 8 days of growth (Figure 4a).

Fungal alpha diversity (Shannon index) in barley sprouts after eight‐day growth was significantly lower than the other two treatments, suggesting the increasing dominance by a few species after eight‐day growth (Figure 4b). Moreover, ordination analysis also confirmed the divergent fungal beta diversity compared between barley seeds and the two time points after seed germination (Figure 4c).

### 3.3 The Proliferation of Fungal Species in Barley Sprouts

**FIGURE 5:**
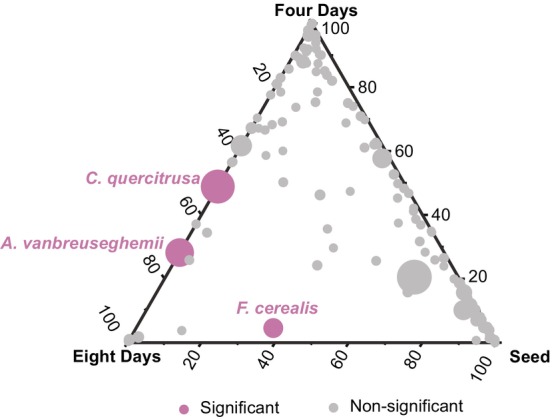
Proliferation of fungal species. The ternary plot compared the relative abundance of all fungal species between barley seeds, four‐day sprouts and eight‐day sprouts. Negative binomial generalised linear model and likelihood ratio test were employed for comparing the fungal relative abundance between treatments. The points represented fungal species and size represented the average relative abundance across all the three treatments. The species with significant proliferation were labelled in pink colour.

To identify the targeting species responsible for the fungal contamination, we compared the relative abundance of fungal species between barley seeds, four‐day sprouts and eight‐day sprouts. In the ternary plot, the abundance of most fungal species showed no significant change in the three treatments except for three species that exhibited significantly higher abundance in eight‐day barley sprouts than in other treatments, which were *C. quercitrusa* and two common fungal pathogens, *A. vanbreuseghemii* and *F. cerealis* (Figure 5).

**FIGURE 6:**
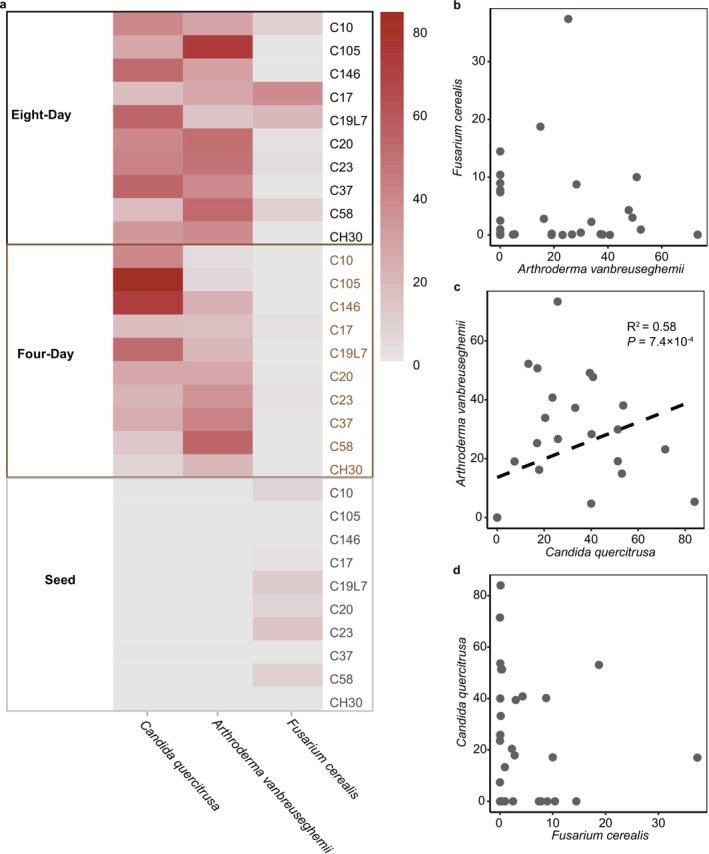
Three fungal species proliferated in eight‐day barley sprouts. (a) Relative abundance of the three fungal species in 10 barley cultivars. (b–d) Correlations between the relative abundance of the three fungal species.

Both growth time‐points (*p* = 1 × 10^−4^, *R* ^2^ = 0.45) and barley cultivars (*p* = 1 × 10^−4^, *R* ^2^ = 0.31) significantly affected the composition of the three 8‐day‐proliferated fungal species (PERMANOVA). After eight‐day growth, the fungal contamination was dominated by *A. vanbreuseghemii* in several species, topped by the 73.4% relative abundance in the C105 cultivar and bottomed by the 14.9% relative abundance in the C19L7 cultivar (Figure 6a). In comparison, *F. cerealis* was the major fungal contamination in C17 (37.4%) and C19L7 (18.7%) cultivars, and the lowest relative abundance of *F. cerealis* was observed in C105 (0.04%) and C37 (0.06%) cultivars (Figure 6a).

Considering that the non‐Saccharomyces yeast *C. quercitrusa* was usually not recognised as a fungal pathogen compared with the other proliferated fungal species, we were interested in its role in the fungal communities. Therefore, we modelled the relationships between the three proliferated species and found that the relative abundance of *C. quercitrusa* was significantly correlated with that of *A. vanbreuseghemii* (*R* ^2^ = 0.58, *p* = 7.4 × 10^−4^) but no other significance was detected (Figure 6b–d).

### 3.4 Treatment of Fungal Contamination in Barley Sprouts

**FIGURE 7:**
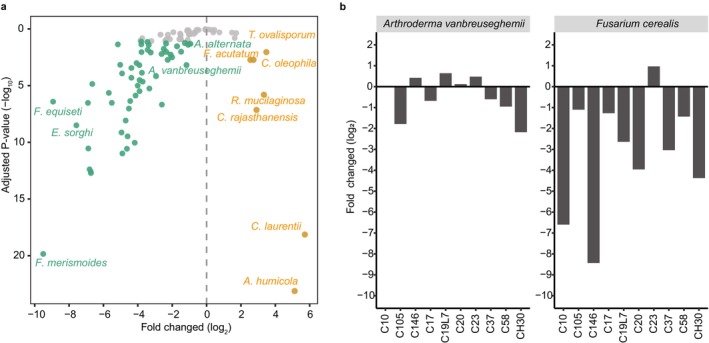
Effect of ozone‐water on the fungal communities in eight‐day barley sprouts. (a) Positive and negative fold changes (log_2_) of normalised read counts represented the change of fungal abundance in ozone‐water treatment relative to the untreated barley sprouts. Significant proliferation (yellow colour) and deficit (green colour) were shown. Grey points represented no significance. (b) Fold change (log_2_) of two major fungal pathogen of *Arthroderma vanbreuseghemii* and *Fusarium cerealis*.

An ozone water generator was used for the control of fungal outbreak in the indoor‐growing barley sprouts. After four‐day treatment of ozone water, we compared the changes of relative abundance in all fungal species. Notably, 55 fungal species were significantly proliferated in the eight‐day sprout without ozone treatment, including the previously mentioned *A. vanbreuseghemii* and several *Fusarium* spp. (Figure 7a). In contrast, seven fungal species were promoted by the ozone treatment, featured by five non‐Saccharomyces yeast species of *Asterotremella humicola*, *C. laurentii* , *C. rajasthanensis*, *Rhodotorula mucilaginosa* and *C. oleophila* (Figure 7a). The effect of ozone‐water treatment varied among barley cultivars. Notably, the read counts (−log_2_ transformed) of *A. vanbreuseghemii* showed the greatest reduction in CH30 and C105 cultivars, whereas *F. cerealis* exhibited the most pronounced decline in C146, C10 and CH30 cultivars following treatment (Figure 7b).

### 3.5 Identification of VOC Markers During Mould Treatment

**FIGURE 8:**
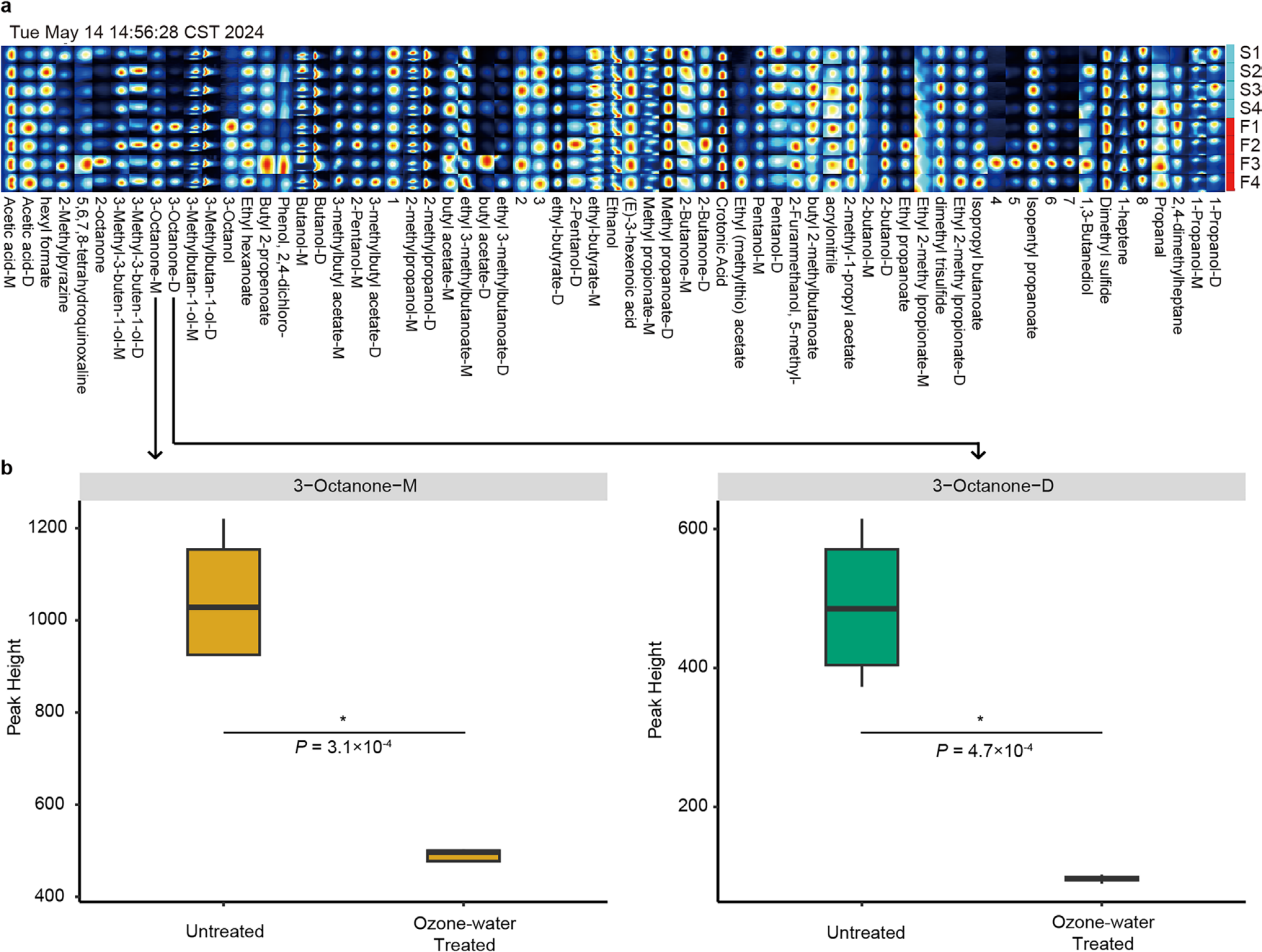
Comparison of volatile organic compounds (VOCs). (a) Comparison of all detected VOC profiles. (b) Signal intensity of Octanone‐M and 3‐Octanone‐D. Both ozone‐water‐treated samples (S1–S4) and non‐treated ones (F1–F4) were detected by GC‐IMS. The intensity of the spot colour correlates with the signal intensity of the volatile compounds, where darker spots indicate higher quantities. Within the matrix, each row encompasses all detected signal peaks for a given sample, whereas each column aligns the signal peaks corresponding to the same VOC across multiple samples.

To fast track the processes of mould outbreak during barley sprouting, we compared the VOC profiles between the C105 barley sprouts treated with ozone water and the untreated ones. Ethanol, Butanol‐D, Crotonic Acid, Acetic acid‐M and 3‐Methylbutan‐1‐ol‐D were the most abundant VOCs (Figure 8a). Through Bonferroni correction of *p* values (*p* < 0.0007), we identified two isomers, 3‐Octanone‐M and 3‐Octanone‐D, which exhibited consistently lower peak intensities in barley sprouts treated with ozone water than the non‐treated ones, suggesting the increase of these compounds was associated with mould outbreak in barley sprouts (Figure 8b).

## 4 Discussion

The current study presents a comprehensive examination of the fungal communities associated with barley sprouts, a critical component in the sprouts industry due to their nutritional value for livestock. Through the application of ITS sequencing, we have identified a diverse array of fungal taxa within barley seeds, with *Cryptococcus* and *Rhodotorula* genera being the most prevalent. Notably, our findings reveal a significant post‐germination shift in the composition of these communities, with species such as *A. vanbreuseghemii*, *F. cerealis* and *C. quercitrusa* demonstrating a marked increase in relative abundance in barley sprouts compared to their initial levels in seeds. This dynamic transition underscores the intricate ecological interplay between fungal species and their host, influenced by both intrinsic factors, such as the genetic constitution of barley cultivars and extrinsic factors, including the controlled environmental conditions of the growth chamber. The observed post‐germination changes highlight the need for a nuanced understanding of the fungal ecology within the context of barley sprout production, as these shifts can have profound implications for both the quality of the barley sprouts and the health of the animals that consume them.

The examination of seed fungal communities across 10 barley cultivars revealed a diverse and complex microbiome. Notably, the genera *Cryptococcus* and *Rhodotorula* contributed a wide range of species to the barley seed mycobiome, highlighting the prevalence of non‐Saccharomyces yeast species in these seeds (Casas‐Godoy et al. 2021). The fungal composition varied among the barley cultivars, which was characterised by differences in the relative abundance of key species, including the pathogenic *Alternaria alternata* (Myo et al. 2019) and *Fusarium cerealis* (Henriksen and Elen 2005). These fungal species have also been recognised as fungal endophytes in different plant hosts (Rashmi et al. 2019). Understanding the initial seed fungal communities is vital, as the presence of a diverse fungal community in the seeds could influence the dynamics of fungal growth during the sprouting process, potentially affecting the quality and safety of the final product intended for animal feed. Our results reveal the different fungal profiles in barley cultivars, suggesting the necessity for selecting barley cultivars to minimise the risk of fungal contamination in the production of barley sprouts.

The observed shift in fungal composition from barley seeds to barley sprouts is a pivotal finding of our study, with significant ecological and agricultural implications. Notably, the emergence of *A. vanbreuseghemii* and *C. quercitrusa* in germinated sprouts suggests a potential adaptation of these species to the dynamic conditions encountered post‐germination. *A. vanbreuseghemii* showed a substantial increase in relative abundance, indicating a possible competitive advantage in the sprout microenvironment. *A. vanbreuseghemii*, known for its ability to infect animals and humans (Chollet et al. 2015), is also found in soil and can be stimulated by elevated levels of carbon and nitrogen fertilisers (Tauro et al. 2021). *C. quercitrusa*, whereas less recognised as a pathogen, also exhibited a significant increase in barley sprouts. Therefore, it warrants further investigation into the ecological function of non‐Saccharomyces yeasts like *C. quercitrusa* within the barley sprouts. Additionally, we showed that the relative abundance of *C. quercitrusa* was positively correlated with that of *A. vanbreuseghemii*. As the relative abundance of these two species increased during the growth of barley sprouts, we speculated that their positive correlation reflected their increased dominance over the whole fungal community. This dominance can also be proved by the results of decreased fungal alpha diversity during barley sprouting. The lack of significant correlations among other dominant species (Figure 6b,d) suggests potential competitive exclusion dynamics, wherein limited resources may favour the dominance of the most well‐adapted species under specific environmental conditions (Dutt et al. 2022). The non‐Saccharomyces yeast *C. quercitrusa* has been identified as a potent biocontrol agent against an oomycete pathogen through producing volatile 2‐Phenylethanol (Lu et al. 2023), which is also observed in many other non‐Saccharomyces yeasts like *Hanseniaspora uvarum* and *Metschnikowia pulcherrima* (Leyva Salas et al. 2017). Therefore, it is possible that *C. quercitrusa* can compete with *A. vanbreuseghemii* during barley sprouting through producing chemical agents. *F. cerealis* was also one of the three proliferated fungal species in the eight‐day sprouts. *Fusarium* species, including *Fusarium cerealis*, are common plant pathogens that cause significant harm to cereal crops, such as wheat, barley and oats (Leyva Salas et al. 2017; Rashmi et al. 2019), underlining their prevalence and potential threat to agricultural systems.

The shift in fungal composition could be indicative of a successional pattern in the fungal community, where early colonisers are replaced by those better adapted to the changing conditions as the sprouts develop. These results were consistent with the observations of changed fungal communities during barley growth (Sapkota et al. 2023) or the industrial malting process (Li et al. 2018). An imbalance in the fungal community, particularly an increase in potential pathogens, could lead to spoilage, reduced nutritional value and even contamination with mycotoxins, posing a risk to livestock health and potentially to the human food chain through dairy and meat products. Therefore, our results demonstrated the need for developing targeted strategies to manage fungal populations and maintain the quality and safety of barley sprouts in the sprouts industry.

Our study proved the effectiveness of ozone water as a treatment to control fungal contamination in barley sprouts, with a focus on its impact on fungal composition. The results indicated that the 2 ppm ozone water treatment significantly reduced the abundance of potentially pathogenic species, including *A. vanbreuseghemii*, *A. alternata* , and several *Fusarium* spp. A previous study demonstrated that ozone water effectively inhibits fungal growth in a concentration‐dependent manner. Specifically, treatment with 2 ppm ozone water resulted in greater suppression of fungal proliferation compared to lower concentrations (1 and 0.5 ppm). Importantly, this higher concentration did not adversely affect barley growth, highlighting the dual efficacy of ozone water as both an antifungal agent and a plant‐compatible treatment (Dai et al. 2024). The potential benefits of using ozone water are multifaceted. Ozone is known for its strong antimicrobial properties through inhibiting fungus spores in plant seeds and seedlings (Kang et al. 2015; Romeo‐Oliván et al. 2022). Its use as a treatment could reduce the reliance on chemical fungicides, aligning with the growing demand for organic and chemical‐free agricultural practices. Moreover, ozone water treatment is considered environmentally friendly, as it decomposes into oxygen, leaving no harmful residues (Gonçalves 2016).

Ozone water treatment also led to an increase of certain fungal species, featured by five non‐Saccharomyces yeasts, which were three *Candida* spp., *Asterotremella humicola and* *Rhodotorula mucilaginosa* . These yeasts were also detected in the barley seed mycobiome in our results, suggesting they may have originated from the seeds rather than being introduced during sprouting. One possible explanation for their proliferation is that ozone water treatment, whereas effectively suppressing filamentous fungi such as *A. vanbreuseghemii* and *Fusarium* spp., may have reduced interspecific competition, thereby creating ecological space for these yeasts to expand. This shift in community composition reflects the selective antifungal effect of ozone, which could indirectly favour the growth of certain tolerant or less susceptible fungi. Given that many of these yeasts have been previously reported to possess biocontrol properties (Casas‐Godoy et al. 2021), their increased abundance after ozone treatment may represent an unintended but potentially beneficial outcome that warrants further investigation in the context of integrated fungal management.

The potential pathogenic *F. acutatum* was also promoted by the ozone water treatment, which may be derived from the adverse effects of ozone water on different fungal species. Consistently, a study showed that certain *Fusarium* species become prevalent after fungicide treatment (Henriksen and Elen 2005). Thus, the development of alternative control measures also warrants further investigation, including beneficial microorganisms as biological control agents. Additionally, it must be noted that our analyses and discussion were based on the relative abundance of the fungal community, and thus the overall fungal biomass may have been altered by the ozone treatment.

The variation in fungal community composition among barley cultivars reveals differential susceptibility to specific pathogens, likely driven by cultivar‐specific biochemical traits that influence microbial recruitment or defence responses. For instance, the predominance of *A. vanbreuseghemii* in C105 (73.4%) and its reduced presence in C19L7 (14.9%), alongside the contrasting abundance of *F. cerealis* in C17 (37.4%) and C19L7 (18.7%) but near absence in C105 and C37, illustrate cultivar‐dependent pathogen affinities. Notably, ozone‐water treatment further modulates these interactions, as evidenced by the marked reduction of *A. vanbreuseghemii* in CH30 and C105 and *F. cerealis* in C146, C10 and CH30. These cultivar‐specific responses may be associated with differential expression of antioxidant enzymes or cell wall modifications, as seen in other ozone‐responsive cereals (Sarkar et al. 2015; Ma et al. 2024). Together, these findings highlight the importance of integrating cultivar‐specific microbial dynamics and ozone responsiveness into the selection and management strategies for indoor barley sprout cultivation.

We also identified that 3‐Octanone isomers showed higher abundance in mouldy barley sprouts than the ones treated with ozone water, suggesting these compounds may serve as indicators of mould outbreak. Several other studies also observed the accumulation of 3‐Octanone during fungal contamination in plants and animals (Ponce et al. 2021; Tian et al. 2023). However, because we have not yet isolated the predominant fungal species from our barley sprouting system, we did not conduct VOC profiling on pure fungal cultures. Therefore, we cannot conclusively determine whether the detected 3‐Octanone isomers originate solely from fungal metabolism, from plant tissue, or from plant‐fungal interactions. Future work will focus on isolating major fungal taxa (e.g., *Arthroderma vanbreuseghemii*, *Fusarium cerealis*) and analysing their VOC emissions in pure culture to verify fungal‐specific production of these markers. Our results highlight the potential of VOC analysis as a tool for rapid detection of mould‐related risks and support the use of ozone water as a viable strategy for mould prevention. 3‐Octanone has emerged as a reliable biomarker for fungal infestation, exhibiting a strong correlation with both infestation duration and fungal activity in stored brown rice grains (Tian et al. 2023). Mechanistically, it is produced via fungal lipid peroxidation pathways and is associated with the regulation of conidium formation, highlighting its role in fungal development and pathogenicity (Speckbacher et al. 2021). Nonetheless, we recognise that our root VOC profiles may reflect a composite of sources, including endogenous plant emissions, microbial metabolism and ozone‐driven oxidation products. Additionally, both root and shoot tissues can contribute to distinct VOC profiles (Voyard et al. 2024). In this investigation, we did not perform source‐resolution experiments. Future studies will analyse VOCs from different plant tissues, profile volatiles emitted by isolated fungal taxa in pure culture, and include ozone‐water‐only controls. These steps will allow us to definitively attribute candidate VOC markers to their origins and refine their use in early fungal outbreak detection. As a volatile organic compound (VOC), 3‐octanone exemplifies the potential of VOCs as non‐invasive indicators for detecting fungal contamination in agricultural systems. These compounds serve as specific biomarkers for pathogen presence and host‐pathogen interactions, thereby enabling early diagnosis, guiding timely intervention strategies, and potentially reducing reliance on chemical fungicides by offering insights into microbial dynamics (Morath et al. 2012). Despite their promise, the application of VOCs in fungal disease monitoring is currently limited by gaps in understanding their biosynthetic pathways, variability in VOC emissions influenced by environmental and interspecies factors, and a lack of comprehensive studies on their ecological functions. Continued research is essential to optimise VOC‐based diagnostics for real‐time and effective disease surveillance (Gan et al. 2023). Finally, while we exclusively profiled VOCs in cultivar C105 due to its pronounced mould outbreak, we recognise that different barley genotypes and their associated fungal communities may emit distinct VOC signatures. Future work will expand VOC profiling across multiple cultivars to validate whether 3‐octanone (and other candidate markers) reliably indicates mould across diverse genetic backgrounds.

## 5 Conclusions

In conclusion, this study provides a comprehensive insight into the complex fungal communities inhabiting barley seeds and their transformation during the germination process into sprouts. The marked shift in fungal populations, with specific species like *A. vanbreuseghemii*, *F. cerealis* and *C. quercitrusa* increasing in abundance, emphasises the dynamic nature of the barley sprout mycobiome and its sensitivity to both genotypic and environmental factors. Our findings underscore the importance of considering barley cultivar selection and managing growth conditions to mitigate fungal contamination risks and ensure the production of high‐quality, safe feed for livestock. The discovery of ozone water as an effective treatment for reducing pathogenic fungi, whereas also highlighting the complexity of intervention impacts on fungal ecology, opens avenues for more sustainable and eco‐friendly approaches in agriculture. However, the unintended proliferation of certain fungi, including potential pathogens like *F. acutatum*, necessitates cautious application and further research into integrated control strategies. Overall, this study contributes to a deeper understanding of fungal community dynamics in barley sprouts and lays the groundwork for future studies aimed at optimising sprout production practices and safeguarding the health of animals and humans in the food chain.

## Author Contributions

**Dedong Kong:** formal analysis, investigation, visualization, writing – original draft. **Mengdi Dai:** investigation, software, funding acquisition, writing – original draft. **Ziran Ye:** software, formal analysis, writing – review and editing. **Yu Luo:** software, formal analysis, writing – review and editing. **Xuting Chen:** investigation, methodology, writing – review and editing. **Xiangfeng Tan:** conceptualization, methodology, project administration, writing – review and editing.

## Conflicts of Interest

The authors declare no conflicts of interest.

## Data Availability

ITS sequencing data is available at Genome Sequence Archive (GSA) database under the accession number of CRA022059.
